# Difference in HIV prevalence by testing venue: results from population level survey in Uganda

**DOI:** 10.1080/09540121.2020.1734179

**Published:** 2020-03-04

**Authors:** Joseph Ouma, Caroline Jeffery, Joseph J. Valadez, Rhoda K. Wanyenze, Jim Todd, Jonathan Levin

**Affiliations:** 1 Division of Epidemiology and Biostatistics, School of Public Health, University of Witwatersrand, Johannesburg, South Africa; 2 METRe Group, Department of International Health, Liverpool School of Tropical Medicine, Pembroke Place, Liverpool L3 5QA, UK; 3 Department of Disease Control and Environmental Health, Makerere University School of Public Health, Kampala, Uganda; 4 Department of Population Health, Faculty of Epidemiology and Population Health, London School of Hygiene and Tropical Medicine, London, United Kingdom

**Keywords:** Population Survey, Health Information System, HIV, Prevalence, Testing Venue

## Abstract

While countries now have national and regional measures of HIV prevalence, sub-regional (district) and sub-district level information is sparse. Growing demand to fill this gap with health facility testing data, in addition to other HIV testing data requires understanding the comparability of these various data sources. We analysed the 2011 Uganda AIDS indicator survey (UAIS) data to assess the proportion of people tested for HIV across Uganda and the venue of testing. We compared HIV prevalence between those tested in a health facility and those testing in a community setting and investigated factors associated with HIV positivity in each subgroup.

We computed HIV prevalence among those tested in a health facility and community setting and obtained HIV prevalence ratio and 95% confidence intervals using the Katz et al (1978) methodology. Factors associated with HIV positivity in each subgroup were assessed using multilevel logistic regression.

Of the 11, 685 individuals, 8,978 (77.1%) had ever tested for HIV in a health facility (female: 6,396, 84.0% versus male: 2,582, 64.2%). Fifty nine percent tested in a health facility in the 12 months preceding the survey (female: 5,507, 72.7% versus male: 1,413, 34.9%). HIV prevalence ratio was1.8 times among those tested in a health facility compared to those tested at community setting (10.9% [95% CI: 10.0-11.7] versus 6.2% [95% CI: 5.4-7.0]). Among heath facility testers, older age group, previously married and having no sexual partner was associated with significantly higher HIV prevalence.

Using facility testing data for program planning and implementation should take into consideration the elevated and varying HIV prevalence among individuals accessing HIV testing services at health facilities as well as differences in their social demographic characteristics.

## Introduction

Population sero-surveys and facility-based HIV testing including testing during antenatal care attendance provide the main sources of data for monitoring the HIV/AIDS epidemic. National population surveys are used to generate accurate estimates of population level HIV/AIDS indicators at national or regional levels while antenatal and other health facility based testing data are used to obtain HIV/AIDS indicator estimates at district level although they possess biases including reporting on only individuals who access health facilities (Eaton et al., 2014; Fabiani, Fylkesnes, Nattabi, Ayella, & Declich, 2003; Gregson, S. Dharmayat et al., 2015; Musinguzi et al., 2009; Wilson et al., 2017).

Hybrid approaches use these data sources to complement each other and hence overcome the limitations of using them independently, to obtain HIV prevalence estimates. The use of hybrid approaches however, requires knowledge of the comparability of the data sources. Population based surveys, contain information on HIV testing including testing venues, that can be analysed to determine how HIV testing data from health facilities can be used to complement data from population survey or other sources to obtain more accurate indicator estimates.

HIV Testing Services (HTS) and approaches have evolved overtime and are broadly categorized as health facility and community-based (Ministry of Health Uganda, 2016; Uganda AIDS Commission, 2017; UNAIDS, 2016). In Uganda, community based HIV testing refers to testing offered in homes, social gatherings/events, in educational establishments and at workplaces, to individuals who do not access health facilities whereas health facility-based HIV testing (Provider-Initiated Testing and Counselling (PITC)) is offered at health facilities as part of health care(Ministry of Health Uganda, 2016).

World Health Organization (WHO) developed the first policy to guide health facility based testing in 2007 (World Health Organization. & Joint United Nations Programme on HIV/AIDS., 2007). By 2012, more than 42 (~80%) of the 52 countries in Africa had adopted it in their national HTS policies (Lule, Granich, & Hargreaves, 2019). In Uganda, PITC is implemented in all hospitals, Health Centre (HC) IV, HC III and in more than 30% of HC II (Uganda AIDS Commission, 2017). See appendix 1 for more details regarding services provided by level of health centre.

Several studies have found higher HIV prevalence and linkage to care among individuals tested at a health facility compared to those tested in a community setting (Govindasamy et al., 2015; Leon et al., 2014; Lugada et al., 2010; Montoy, Dow, & Kaplan, 2016; Roura, Watson-jones, Kahawita, Ferguson, & Ross, 2013; Wanyenze et al., 2008, 2009), and others have found similar HIV prevalence estimates for community based testing during a population surveys and those tested in health facility during antenatal care (Gonese et al., 2010; Judith RG, Anne B, Michel C, Rosemary MM, Maina K, Isaac M, Francis T, 2001; Musinguzi et al., 2009; Wilson et al., 2017).

We analysed data from the 2011 UAIS to address two questions. First, what proportion of people have tested for HIV across Uganda, and where have they tested? Second, how does the HIV prevalence compare between those who have tested in a health facility and those who have tested in a community setting. We also investigate factors associated with HIV positivity in each of these subgroups.

## Methods

During the UAIS 2011 survey, the country was divided into 10 geographical regions. Survey sample sizes were allocated equally across regions. Clusters were randomly selected from each region with probability proportional to number of households in a cluster (Ministry of Heath and ICF international, 2012). A systematic sample of 25 households was selected from each cluster and all adults present in the selected households who consented to participate in the survey were interviewed and blood drawn for HIV testing(Ministry of Heath and ICF international, 2012). More details about the survey are available from https://dhsprogram.com.

We analysed data of 11,685 individuals aged 15-49 years, who reported having “*ever tested”* for HIV before the survey.

During the survey, respondents were asked “*Have you ever been tested to see if you have the AIDS virus?*”, if they answered “*yes*”, additional questions relating to period of the most recent test, and receipt of test results were asked. Women were asked questions relating to antennal attendance; “*Were you offered a test for the AIDS virus as part of your antenatal care?*” if they answered yes, additional questions regarding place of testing and receipt of test results were asked.

### Study variables

(1) *HIV status.* HIV testing was conducted for all consenting individuals and results provided to the respondent at home. Only final test results (either 1=Positive or 0=Negative) were provided.
(2) *Testing venue (1=Health facility or 0=community setting):*
*Tested in a health Facility:* Individuals who had ever tested for HIV in a health facility and received test results prior to the survey, including women who tested during antenatal care. Health facilities include hospitals; public HC II, III and IV; private hospitals/clinics; and organisations offering HIV/AIDS care and treatment services.
*Tested in a community setting:* Individuals ever tested during community events, at their homes or work places before the survey. It also includes individuals who were tested in standalone voluntary counselling and testing centres not offering general healthcare.
(3) *Explanatory/independent variables*: area of residence (1=urban, 2=rural), gender (1=male, 2=female), age group (15-19, 20-29, 30-39 and 40-49-years), marital status (never married, married/cohabiting and previously married-widowed/separated/divorced), highest level of education (none, primary, secondary or higher), number of sexual partners including husband/wife in the 12 months preceding the survey (0, 1 and 2 or more), employment status (employed, not employed), and distance to nearest health facility in kilometres (categorised into <2, 2-5, 5 or more).

### Statistical analysis

We computed the proportion tested for HIV; prevalence by venue of testing; and HIV Prevalence Ratio (PR) and the 95% confidence intervals for health facility compared to community testing using the Katz methodology (Appendix 2) (Azen S.P., 1978; Koopman, 1984). We also assessed factors associated with HIV positivity in each subgroup using multilevel logistic regression. We further compared HIV prevalence among those tested in a health facility and community; a) overall and b) among those tested in the 12 months preceding the survey. All analysis was carried out using Stata release 15 (StataCorp, 2017) and weighted by population sampling weights.

## Results

Of the 11,685 (female: 7,647, 65.1%) individuals, 4,789 (41.3%) were aged 20-29 years, 1,216 (9.9) % had no formal education, 1,375 (11.1%) were previously married and 1,097 (9.7%) reported that they had two or more sexual partners in the 12 months preceding the survey.

Of those who tested in a health facility, 6,396 (70.9%) were female, 3,894 (43.5%) were aged 20-29 years, 6,609 (73.7%) were married/cohabiting and 6,980 (77.4%) had only one sexual partner. Of the 2,707 tested for HIV in a community setting, 1,251 (45.3%) were female, 895 (33.9%) were aged 20-29 years, 1,560 (59.0%) were married or cohabiting while 1,669 (62.8%) had one sexual partner (Table 1).

### Prevalence of ever testing by venue of testing

Of the 11,685 individuals, 8,978 (77.1%) had tested for HIV in a health facility (female: 6,396, 84.0% versus male: 2,582, 64.2%) (Appendix 3). Testing in a health facility was higher among individuals aged 20-29 years (3,894, 81.2%), no education (1,007, 82.3%), married or cohabiting (6,609, 80.8%) and among those with one sexual partner in the 12 months preceding the survey (6, 980, 80.6%). In contrast, testing in a health facility was lower among males (2,582, 64.2%), age group 15-19 years (953, 65.1%), never married (1,301, 61.4%) and among those who had no sexual partners in the 12 months preceding the survey (1,252, 65.4%) (Appendix 3).

### HIV Prevalence by venue of testing

HIV prevalence was 9.9% (95% CI: 9.2-10.7) and 5.8% (95% CI: 4.8-6.8) among those tested in a health facility and in a community setting respectively (Table 2). Prevalence among those tested in a health facility was highest in age group 40-49 years (15.0%, 95% CI: 12.9-17.1); previously married 25.7% (95% CI: 22.6, 28.7); and in those who had no sexual partner in the 12 months preceding the survey (15.7%, 95% CI: 13.4-18.1) (Table 2). Prevalence among those tested in a community setting was higher among previously married and those who had 2 or more sexual partners in the 12 months preceding the survey (8.2%, 95% CI: 5.1-11.3).

### HIV prevalence by testing venue among those who tested in the 12 months preceding survey

Of the 11,685 individuals included in the analysis, 6,920 (59.2%) tested in a health facility in the 12 months preceding the survey (female: 5,507, 72.7% versus male: 1,413, 34.9%). HIV prevalence among health facility testers was 10.9% (95% CI: 10.0-11.7) compared to 6.2% (95% CI: 5.4-7.0) among individuals tested in a community setting (Table 3).

HIV PR was 1.8 (95% CI: 1.6, 2.1) for those tested in a health facility compared to those tested in a community setting. PR was 3.8 (95% CI: 2.4, 6.0) for those who had no sexual partner in the 12 months preceding the survey; 2.7 (95% CI: 1.9, 3.9) among males; 3.2 (95% CI: 1.3, 8.0) for age group 15-19 years; 2.4 (95% CI: 1.3, 4.5) for those with no education; and 2.2 (95% CI: 1.2, 4.2) for never married (Table 3).

Among health facility testers, the odds of testing HIV positive during the survey was significantly higher in age group 40-49 years compared to 20-29 years (aOR; 2.92, 95% CI: 2.28-3.73); previously married versus married/cohabiting (aOR; 3.25, 95% CI: 2.66, 4.12); and for those who had no sexual partner versus those who had one (aOR; 1.55, 95% CI: 1.20, 2.00). HIV positivity was significantly lower among females compared to males (aOR: 0.78, 95% CI: 0.62, 0.97); age group 15-19 compared 20-29 years (aOR: 0.67, 95% CI: 0.45, 0.99); secondary versus primary education (aOR: 0.64, 95% CI: 0.52, 0.80) and rural versus urban residence (aOR; 0.59, 95% CI: 0.43, 0.80) (Table 4).

For those tested in a community setting, the odds of testing HIV positive was significantly higher for females compared to males (aOR; 1.62, 95% CI: 1.21, 2.15); previously married versus married/cohabiting (aOR: 2.05, 95% CI: 1.44, 2.92); and among those who had two or more sexual partners compared to those who had one (aOR; 1.95, 95% CI: 1.37, 2.76). The likelihood of a positive test result was significantly lower among those aged 15-19 years versus age group 20-29 years (aOR: 0.34, 95% CI: 0.17, 0.70) (Table 4). Further analysis comparing HIV prevalence among those tested in a health facility in 12 months preceding the survey with those tested in a community setting are presented in appendix 4.

### Prevalence of testing for HIV in a health facility in the 12 months preceding survey

Prevalence of testing for HIV in a health facility in the 12 months preceding the survey was higher among females (5,507, 72.7%); age group 20-29 years (3,221, 67.1%); married/cohabiting (5,204, 63.7%); and in those who reported one sexual partner in the 12 months preceding the survey (5,585, 64.8%). Testing was lower among males (1,413, 34.9%); age group 15-19 years (724, 50.1%); age group 40-49 (866, 43.4%); never married (891, 42.5%); and among those who had two or more sexual partners in the 12 months preceding the survey (463, 43.2%) (Table 3).

## Discussion

We analysed data from 2011 UAIS, to estimate the proportion of individuals ever tested for HIV; prevalence by testing venue; HIV PR for those tested in a health facility to those tested in a community setting; and assessed factors associated with HIV positivity for each subgroup

Findings show that 59.0% of the respondents tested for HIV in a health facility in the 12 months preceding the survey. Overall HIV PR was 1.8. PR was 2 or more times higher among males; age groups 15-19, 30-39 and 40-49 years; those with no education; never or previously married; and among those who had no sexual partner in the 12 months preceding the survey.

Higher HIV prevalence among health facility compared to community based testing found in this study is consistent with findings elsewhere (Chirawu et al., 2010; Govindasamy et al., 2015; Lugada et al., 2010; Sharma, Ying, Tarr, & Barnabas, 2015; Silvestri et al., 2011). For example, in a rural community in East Central Uganda, HIV prevalence was 2.7 (17.3% vs. 7.1%) times higher in the clinic-based compared to home-based arm (Lugada et al., 2010) while in Zimbabwe, positivity rates were 32.9% and 18.8% for clinic and community based arms respectively (Chirawu et al., 2010). These findings demonstrate higher HIV prevalence (~2-fold) in health facility testing compared to prevalence based on HIV testing from a community setting.

Furthermore, HIV prevalence was 3.6% (10.9% vs 7.3%) higher and 1.9% (5.4% vs 7.3%) lower among individuals tested in health facility and those tested in a community setting respectively compared to overall prevalence in the general population (Ministry of Heath and ICF international, 2012).

Higher HIV prevalence among health facility compared to community based testing have been attributed to ill health among individuals accessing health facilities in many studies (Chirawu et al., 2010; Sharma et al., 2016; Silvestri et al., 2011; Ssebunya et al., 2018). Other studies attributed higher HIV prevalence among health facility testers to a desire by individuals to know status after a risky sexual behaviour (Ssebunya et al., 2018).

In this study, we observed similar HIV prevalence (PR=1.1) for those tested at a health facility compared to those tested at community setting for the age group 20-29 years. This age group comprise mainly newly married individuals who have a desire to have children and are therefore more likely to visit a health facility for antenatal or child health reasons and not a recent high-risk sexual behaviour or ill health. Similarly, HIV PR for married/cohabiting individuals and those who had one sexual partner was close to 1, (i.e. 1.3, and 1.5 respectively). These population sub groups are more likely to be in stable sexual/family relationship and therefore are more likely to visit a health facility for antenatal or family health reasons and thus less likely to test HIV positive. Additionally, in sub-Saharan Africa, caring for sick family members are often delegated to women who attend to/take care of sick family members seeking health care. This phenomenon may explain the PR (~1), observed among females who had tested at health facilities compared to those tested at a community setting.

Several studies have also found lower fertility among HIV positive women compared to their HIV negative counterparts (Gregson S, Terceiria N, Kakowa M, Mason PR, Anderson RM, Chandiwana SK, 2002; Zaba et al., 2000). This implies that women who visit health facilities for antenatal reasons are less likely to test HIV positive, similar to findings in this study.

Additionally, this study found higher HIV PR among never married individuals; previously married; and those with no sexual partners tested at health facilities compared those tested at a community setting. This may be attributed to ill health or a recent risky sexual behaviour in these population subgroups as noted above.

Regarding factors associated with HIV positivity, similar factors were found for those tested in a health facility and those testing in a community setting for all socio demographic characteristics except gender. Among those tested in a health facility, HIV positivity was significantly lower among females compared to males. However, positivity was significantly higher among females tested in a community setting compared to males consistent with findings elsewhere (Amornkul et al., 2009; Kwesigabo et al., 2000; World Health Organization, 2015; Zaba et al., 2000). Lower positivity rates among females tested at health facilities compared to males may be attributed to males seeking care at health facilities due to ill health, while women may visit health facilities for antenatal or other family health reasons as noted above.

We also observed higher HIV positivity rates among individuals who reported no sexual partner compared to those who had only one partner in the 12 months preceding the survey irrespective of the venue of testing. Higher HIV positivity rates in this subgroup may be attributed to concealment of existing or earlier sexual relationships consistent with a study in Uganda, that found never and previously married individuals had more sexual partners compared to those who were married (Nalugoda et al., 2014).

Although prevalence of health facility testing was generally low at 59%, testing was lower among males, individuals who had two or more sexual partners, those with primary or lower level of education consistent with findings from other studies (Amornkul et al., 2009; Manda, Masenyetse, Cai, & Meyer, 2015; Mekonnen, Lerebo, Gebrehiwot, & Abadura, 2015; Mtenga et al., 2015; Mtowa, Gerritsen, Mtenga, Mwangome, & Geubbels, 2017; Muyunda, Musonda, Mee, Todd, & Michelo, 2018; Nalugoda et al., 2014). The proportion of males tested in a health facility was only 34.9% compared to 72.7% of females. In Uganda, there is almost universal HCT coverage among women seeking antenatal and postnatal services (>95%) (Uganda Bureau of Statistics (UBOS) and ICF International Inc, 2012), accounting for higher testing rates among females compared males found in this study. We also observed lower testing rates in age groups 15-19years and 40-49 years consistent with other studies in Sub-Saharan Africa (Staveteig et al., 2017). The low testing rates in older age groups has been attributed to reluctance by health workers to prioritize testing for older age groups and poor health seeking behaviour among youths and older persons (Kiplagat, 2018). Other well documented barriers for HCT access by men and youth include fear to find out ones test results; avoiding to divulge personal information to health workers (Facha, Kassahun, & Workicho, 2016; Mohlabane N, Tutshana B, Peltzer K, 2019).

### Limitations

This study used population survey data, many limitations including, recall of information such as, when and where the last HIV test was conducted may be influenced by the respondent’s personal experiences during the test and whether the experience was desirable. Individuals may prefer not to report negative experiences such as positive HIV test result during a previous test. Reasons for testing for HIV at a health facility or community setting were not captured during the survey.

## Conclusions

We found higher HIV prevalence among individuals who tested in a health facility compared to those tested in a community setting. HIV PR was more than a two-fold in males; age groups 15-19 and 40-49 years; never married and those who had no sexual partners in the 12 months preceding the survey while prevalence of facility testing was lower in these age groups. Higher HIV prevalence among specific population subgroups accessing health facilities and the low testing rates in those population subgroups call for continuous review of Health Management Information System (HMIS) data to inform scaling up of HIV testing interventions. Additionally, HMIS data comprise data from public health facilities only, excluding private health facilities accessed by wealthier and more educated individuals whose HIV prevalence rates may be different from that of the general population.

## Supplementary Material

Appendix

## Figures and Tables

**Table 1:** Socio-demographic characteristics of study participants

	Overall	Tested in Health Facility	Tested in Community
Characteristic	n=11,685 (%)	n=8,978 (%)	n=2,707 (%)
**Gender**			
Male	4,038 (34.9)	2,582 (29.1)	1,456 (54.7)
Female	7,647 (65.1)	6,396 (70.9)	1,251 (45.3)
**Age**			
15-19	1,498 (12.8)	953 (10.8)	545 (19.5)
20-29	4,789 (41.3)	3,894 (43.5)	895 (33.9)
30-39	3,422 (29.3)	2,716 (30.3)	706 (26.1)
40-49	1,976 (16.6)	1,415 (15.4)	561 (20.5)
**Education Level**			
No Education	1,216 (9.9)	1,007 (10.6)	209 (7.7)
Primary	6,406 (55.1)	5,003 (55.9)	1,403 (52.2)
Secondary+	4,063 (35.0)	2,968 (33.5)	1,095 (40.1)
**Marital status**			
Never married	2,159 (18.6)	1,301 (14.8)	858 (31.3)
Married/Cohabiting	8,169 (70.3)	6,609 (73.7)	1,560 (59.0)
Previously married	1,357 (11.1)	1,068 (11.5)	289 (9.8)
**Number of sexual partners in 12 months preceding survey**			
0	1,957 (16.2)	1,252 (13.7)	705 (24.5)
1	8,649 (74.1)	6,980 (77.4)	1,669 (62.8)
2+	1,079 (9.7)	746 (8.9)	333 (12.8)
**Currently working**			
No	2,894 (24.2)	2,219 (24.3)	675 (23.8)
Yes	8,791 (75.8)	6,759 (75.7)	2,032 (76.2)
**Distance to nearest HF**			
<2	2,441 (21.7)	1,920 (22.2)	521 (20.0)
2-5	4,823 (40.7)	3,696 (40.6)	1,127 (40.8)
5+	4,009 (33.7)	3,040 (33.3)	969 (35.2)
Don’t Know	412 (3.9)	322 (3.9)	90 (3.9)
**Area of residence**			
Rural	8,870 (75.8)	6,845 (76.0)	2,025 (75.4)
Urban	2,815 (24.1)	2,133 (24.0)	682 (24.6)
**Region**			
Central 1	1,151 (12.2)	889 (12.3)	262 (12.2)
Central 2	1,177 (10.6)	861 (10.0)	316 (12.8)
Kampala	1,464 (9.0)	1,068 (8.4)	396 (11.0)
East Central	1,013 (8.8)	722 (8.2)	291 (10.5)
Mid-Eastern	901 (7.6)	732 (8.0)	169 (6.3)
North East	1,134 (9.2)	875 (9.0)	259 (10.0)
West Nile	1,220 (6.4)	896 (6.0)	324 (7.6)
Mid Northern	1,394 (12.5)	1,107 (12.9)	287 (11.3)
South Western	1,024 (11.3)	853 (12.2)	171 (8.0)
Mid-Western	1,207 (12.4)	975 (12.9)	232 (10.5)

Note: Percentages are weighted by population survey weights

**Table 2:** HIV prevalence distribution overall and by venue of testing

	Overall (N=11,685)		Ever tested for HIV in*			
			Health Facility			Community
Characteristic	n#	Weighted Prevalence (95% CI)	n#	Weighted Prevalence (95% CI)	n#	Weighted Prevalence (95% CI)
**Total**	1,002	9.0 (8.4, 9.6)	850	9.9 (9.2, 10.7)	152	5.8 (4.8, 6.8)
**Gender**						
Male	300	7.8 (6.8, 8.8)	227	9.4 (8.1, 10.8)	73	4.9 (3.7, 6.1)
Female	702	9.6 (8.9, 10.4)	623	10.2 (9.3, 11.0)	79	6.9 (5.2, 8.5)
**Age**						
15-19	50	3.6 (2.5, 4.8)	42	4.7 (3.1, 6.3)	8	1.7 (0.5, 2.9)
20-29	314	6.9 (6.0, 7.7)	268	7.2 (6.3, 8.2)	46	5.4 (3.7, 7.2)
30-39	382	11.9 (10.6, 13.1)	334	13.2 (11.7, 14.7)	48	6.7 (4.7, 8.8)
40-49	256	13.3 (11.6, 15.0)	206	15.0 (12.9, 17.1)	50	9.0 (6.5, 11.6)
**Education Level**						
No Education	123	10.4 (8.6, 12.3)	112	11.6 (9.4, 13.8)	11	5.0 (1.9, 8.0)
Primary	614	10.1 (9.3, 11.0)	523	11.0 (10.0, 12.0)	91	6.9 (5.3, 8.4)
Secondary	265	6.8 (5.9, 7.7)	215	7.6 (6.5, 8.8)	50	4.5 (3.2, 5.9)
**Marital status**						
Never married	89	4.5 (3.4, 5.6)	69	5.6 (4.2, 7.1)	20	2.7 (1.3, 4.2)
Married/Living together	622	7.9 (7.2, 8.6)	529	8.4 (7.6, 9.1)	93	6.0 (4.7, 7.3)
Previously married	291	23.4 (20.8, 26.0)	252	25.7 (22.6, 28.7)	39	14.3 (9.9, 18.8)
**Number of sexual partners in 12 months preceding survey**						
0	219	11.9 (11.2, 13.5)	189	15.7 (13.4, 18.1)	30	4.5 (2.8, 6.3)
1	672	8.1 (7.5, 8.8)	580	8.7 (7.9, 9.5)	92	5.8 (4.5, 7.1)
2+	111	10.7 (8.6, 12.8)	81	11.8 (9.0, 14.5)	30	8.2 (5.1, 11.3)
**Currently working**						
No	205	7.2 (6.1, 8.3)	178	8.1 (6.8, 9.4)	27	4.1 (2.5, 5.8)
Yes	797	9.6 (8.9, 10.3)	672	10.5 (9.7, 11.4)	125	6.3 (5.1, 7.5)
**Distance to nearest HF**						
<2	222	9.3 (7.9, 10.7)	192	10.1 (8.5, 11.7)	30	6.4 (3.7, 9.1)
2-5	374	8.1 (7.3, 9.0)	316	9.0 (8.0, 10.1)	58	5.2 (3.8, 6.6)
5+	375	9.8 (8.8, 10.8)	315	10.9 (9.7, 12.1)	60	6.3 (4.6, 7.9)
Don’t Know	31	9.0 (5.2, 12.9)	27	10.5 (5.7, 15.3)	4	4.0 (0.0, 8.1)
**Area of residence**						
Rural	737	8.7 (8.1, 9.4)	624	9.6 (8.8, 10.3)	113	5.9 (4.7, 7.0)
Urban	265	9.9 (8.5, 11.3)	226	11.2 (9.5, 12.9)	39	5.5 (3.4, 7.7)
**Region**						
Central 1	144	13.0 (10.7, 15.2)	123	14.3 (11.6, 16.9)	21	8.5 (4.4, 12.5)
Central 2	116	9.9 (8.1, 11.7)	99	11.4 (9.1, 13.7)	17	6.0 (3.1, 8.9)
Kampala	116	7.8 (6.2, 9.3)	94	9.0 (7.0, 11.0)	22	4.4 (2.4, 6.4)
East Central	65	7.1 (5.4, 8.9)	52	8.0 (5.8, 10.2)	13	5.0 (2.2, 7.8)
Mid-Eastern	50	5.4 (3.9, 6.9)	41	5.3 (3.7, 7.0)	9	5.6 (2.0, 9.2)
North East	84	6.7 (5.0, 8.4)	71	7.7 (5.5, 9.8)	13	3.6 (1.5, 5.7)
West Nile	67	6.0 (4.5, 7.5)	54	6.5 (4.7, 8.3)	13	4.8 (2.2, 7.4)
Mid Northern	133	9.7 (7.9, 11.5)	124	11.5 (9.3, 13.7)	9	2.9 (0.9, 4.9)
South Western	110	10.5 (8.5, 12.4)	94	10.9 (8.7, 13.1)	16	8.3 (4.2, 12.4)
Mid-Western	117	9.9 (8.0, 11.7)	98	10.2 (8.1, 12.2)	19	8.6 (4.7, 12.5)

# Number HIV positive, Denominators are presented in table 1. HIV prevalence estimates are weighted by population survey weights

* HIV prevalence ratio comparing health facility and community testers

**Table 3:** Proportion tested for HIV in a health facility in the 12 months preceding the survey and HIV prevalence by testing venue

Characteristic	Tested in Health facility in 12 months preceding the survey				Tested in community setting		HIV Prevalence Ratio (95% CI)
	Number tested (%)		n#	Weighted Prevalence (95% CI)	n#	Weighted Prevalence (95% CI)	
**Total**	**6,920**	**59.0**	**722**	**10.9 (10.0, 11.7)**	**100**	**6.2 (5.4, 7.0)**	**1.8 (1.4, 2.2)**
**Gender**							
Male	1,413	34.9	169	12.6 (10.6, 14.7)	37	4.6 (3.0, 6.2)	2.7 (1.9, 3.9)
Female	5,507	72.7	553	10.4 (9.5, 11.3)	63	7.8 (5.7, 9.9)	1.3 (1.0, 1.7)
**Age**							
15-19	724	50.1	37	5.4 (3.5, 7.4)	5	1.7 (1.9, 3.3)	3.2 (1.3, 8.0)
20-29	3,221	67.1	225	7.2 (6.2, 8.2)	35	6.7 (4.2, 9.2)	1.1 (0.8, 1.5)
30-39	2,109	62.0	286	14.7 (12.9, 16.4)	30	6.3 (3.9, 8.6)	2.3 (1.6, 3.3)
40-49	866	43.4	174	20.3 (17.4, 23.2)	30	10.3 (6.5, 14.0)	2.0 (1.4, 2.8)
**Education Level**							
No Education	782	63.7	98	13.3 (10.7, 15.9)	10	5.5 (1.9, 9.1)	2.4 (1.3, 4.5)
Primary	3,917	61.3	446	12.1 (10.9, 13.2)	60	7.5 (5.4, 9.5)	1.6 (1.2, 2.1)
Secondary	2,221	55.5	178	8.0 (6.7, 9.3)	30	4.8 (3.0, 6.6)	1.7 (1.1, 2.4)
**Marital status**							
Never married	891	42.5	54	6.4 (4.5, 8.2)	11	2.9 (0.8, 5.1)	2.2 (1.2, 4.2)
Married/Cohabiting	5,204	63.7	445	8.8 (7.9, 9.7)	67	6.8 (5.1, 8.5)	1.3 (1.0, 1.7)
Previously married	825	61.5	223	29.7 (26.1, 33.3)	22	13.5 (7.8, 19.1)	2.2 (1.5, 3.3)
**Number of sexual partners in 12 months preceding survey**							
0	872	45.1	166	20.2 (17.1, 23.4)	20	5.3 (2.9, 7.8)	3.8 (2.4, 6.0)
1	5,585	64.8	499	9.3 (8.4, 10.1)	60	6.3 (4.5, 8.1)	1.5 (1.1, 1.9)
2+	463	43.2	57	12.8 (9.4, 16.3)	19	7.7 (4.2, 11.3)	1.7 (1.0, 2.7)
**Currently working**							
No	1,820	64.3	158	8.7 (7.2, 10.2)	21	3.9 (2.1, 5.8)	2.2 (1.4, 3.5)
Yes	5,100	58.0	564	11.6 (10.6, 12.6)	79	7.1 (5.4, 8.8)	1.6 (1.3, 2.0)
**Distance to nearest HF**							
<2	1,493	62.0	171	11.3 (9.4, 13.2)	18	6.2 (2.6, 9.7)	1.8 (1.1, 2.9)
2-5	2,852	59,3	256	9.4 (8.1, 10.6)	34	5.5 (3.5, 7.4)	1.7 (1.2, 2.4)
5+	2,341	58.5	275	12.5 (11.0, 14.0)	44	7.2 (5.0, 9.4)	1.7 (1.3, 2.4)
Don’t Know	234	55.8	20	10.0 (4.8, 15.2)	4	6.0 (0.1, 11.8)	1.7 (0.6, 4.7)
**Area of residence**							
Rural	5,284	59.6	530	10.5 (9.6, 11.4)	77	6.3 (4.8, 7.7)	1.7 (1.3, 2.1)
Urban	1,636	59.2	192	12.1 (10.1, 14.1)	23	6.2 (2.9, 9.5)	2.0 (1.3, 3.0)
**Region**							
Central 1	662	57.5	95	14.8 (11.7, 17.9)	14	9.6 (4.0, 15.2)	1.5 (0.9, 2.6)
Central 2	713	60.2	81	11.0 (8.5, 13.5)	11	6.1 (2.3, 9.9)	1.8 (1.0, 3.3)
Kampala	803	54.1	81	10.3 (7.8, 12.7)	13	5.1 (2.1, 8.1)	2.0 (1.1, 3.6)
East Central	547	55.0	48	9.8 (7.0, 12.5)	8	4.8 (1.4, 8.3)	2.0 (1.0, 4.2)
Mid-Eastern	544	60.5	31	5.4 (3.5, 7.3)	10	7.1 (2.8, 11.4)	0.8 (0.4, 1.5)
North East	711	63.4	62	7.6 (5.4, 9.8)	11	7.5 (2.9, 12.0)	1.0 (0.5, 1.9)
West Nile	680	54.8	47	7.6 (5.3, 9.8)	9	5.3 (1.8, 8.8)	1.4 (0.7, 2.9)
Mid Northern	886	62.8	106	12.5 (9.8, 15.1)	6	2.6 (0.4, 4.9)	4.8 (2.1, 10.8)
South Western	639	62.5	86	13.4 (10.6, 16.2)	9	9.7 (3.2, 16.2)	1.4 (0.7, 2.6)
Mid-Western	735	60.8	85	11.6 (9.1, 14.1)	9	6.3 (2.1, 10.6)	1.8 (0.9, 3.6)

# number HIV positive

**Table 4:** Factors associated with HIV prevalence by venue of testing

Characteristic	Overall				Tested in Health facility		Tested in Community	
	cOR	(95% CI)	aOR	(95% CI)	aOR	(95% CI)	aOR	(95% CI)
**Gender (Reference group: Male)**								
Female	1.25	(1.09, 1.44)*	1.21	(1.03, 1.43)*	0.78	(0.62, 0.97)*	1.62	(1.21, 2.15)*
**Age (Reference group: 20-29)**								
15-19	0.48	(0.35, 0.66)*	0.53	(0.38, 0.75)*	0.67	(0.45, 0.99)**	0.34	(0.17, 0.70)*
30-39	1.86	(1.58, 2.18)*	1.70	(1.43, 2.03)*	1.96	(1.60, 2.42)*	1.24	(0.89, 1.74)
40-49	2.28	(1.90, 2.72)*	1.89	(1.55, 2.31)*	2.92	(2.28, 3.73)*	1.17	(0.80, 1.69)
**Education Level ((Reference group: Primary)**								
No Education	1.09	(0.86, 1.38)	0.86	(0.68, 1.10)	0.91	(0.69, 1.19)	0.70	(0.41, 1.19)
Secondary	0.61	(0.52, 0.72)*	0.72	(0.60, 0.86)*	0.64	(0.52, 0.80)*	0.85	(0.61, 1.18)
**Marital status (Reference Group: Married/Living together**								
Never married	0.49	(0.39, 0.63)*	0.80	(0.58, 1.1)	0.93	(0.64, 1.37)	0.74	(0.43, 1.27)
Previously married	3.33	(2.81, 3.96)*	2.76	(2.27, 3.35)*	3.25	(2.66, 4.12)*	2.05	(1.44, 2.92)*
**Number of sexual partners in 12 months preceding survey (Reference Group: 1)**								
0	1.52	(1.27, 1.81)*	1.32	(1.07, 1.63)*	1.55	(1.20, 2.00)*	1.01	(0.67, 1.53)
2+	1.39	(1.11, 1.74)*	1.43	(1.12, 1.82)*	1.07	(0.77, 1.50)	1.95	(1.37, 2.76)*
**Currently working (Reference Group: Not employed)**								
Employed	1.34	(1.13, 1.61)*	1.02	(0.84, 1.24)	1.03	(0.83, 1.28)	0.99	(0.65, 1.50)
**Distance to nearest HF (Reference Group: <2Km)**								
2-5	0.80	(0.66, 0.98)**						
5+	1.02	(0.83, 1.25)						
Don’t Know	0.79	(0.53, 1.19)						
**Area of residence (Reference Group: Urban)**								
Rural	0.84	(0.69, 1.03)	0.62	(0.47, 0.82)*	0.59	(0.43, 0.80)*	0.75	(0.46, 1.23)
**Region (Reference Group: Central 1)**								
Central 2	0.75	(0.56, 1.02)	0.74	(0.53, 1.02)	0.75	(0.51, 1.10)	0.71	(0.41, 1.21)
Kampala	0.60	(0.43, 0.84)*	0.49	(0.32, 0.75)*	0.53	(0.33, 0.86)*	0.47	(0.23, 0.93)*
East Central	0.45	(0.31, 0.66)*	0.46	(0.31, 0.66)*	0.58	(0.37, 0.90)*	0.30	(0.15, 0.60)*
Mid-Eastern	0.40	(0.25, 0.64)*	0.44	(0.27, 0.71)*	0.37	(0.23, 0.62)*	0.53	(0.24, 1.14)
North East	0.54	(0.38, 0.77)*	0.58	(0.40, 0.82)*	0.56	(0.37, 0.84)*	0.53	(0.30, 0.95)*
West Nile	0.38	(0.26, 0.56)*	0.36	(0.23, 0.56)*	0.38	(0.22, 0.64)*	0.33	(0.18, 0.63)*
Mid Northern	0.72	(0.52, 1.01)	0.80	(0.56, 1.13)	0.78	(0.53, 1.16)	0.59	(0.33, 1.11)
South western	0.82	(0.60, 1.14)	0.87	(0.62, 1.22)	0.93	(0.62, 1.38)	0.65	(0.38, 1.14)
Mid-western	0.73	(0.53, 102)	0.78	(0.55, 1.11)	0.82	(0.54, 1.24)	0.69	(0.40, 1.17)

* p-value<0.01

** P-value<0.05, cOR-unadjusted Odds Ratio, aOR-Adjusted Odds Ratio

## Data Availability

Data used for this study is publicly available from Measure DHS: https://dhsprogram.com
