# Supplementary material for: Difference in HIV prevalence by testing venue: results from population level survey in Uganda
Source: AIDS Care. Author manuscript; Available in PMC 2022 Apr 12. (PMC7612612; doi:10.1080/09540121.2020.1734179)
Supplement: Appendix [file EMS144093-supplement-Appendix.pdf]

Appendix 1: Health Care classification in Uganda

1. Health Center (HC) II is the lowest level of service delivery. It is located at parish level. It forms the first contact with the formal health sector for the community. It provides Primary Health Care (PHC) and outpatient clinical services to about 5,000 people. It has no inpatient and laboratory services and is headed by a nurse.
2. Health Center (HC) III is located in the sub-Counties, provides PHC, laboratory, clinical outpatient as well as maternity services. Its catchment population is 20,000 people. It is a referral facility for the community and HC II.
3. Health Center (HC) IV is located at the county level or sub-district level and covers a population of 100,000 people. It provides PHC, clinical, maternity, laboratory, blood transfusion and emergency surgery services. It is a referral facility for the community, HC II and III.

Appendix 2: Method for computing confidence

Let X and Y be independent binomial variates based on the sample sizes m and n and parameters $p_1$and p_2, respectively. Let the $\theta = p_1/p_2$, We computed the prevalence ratio confidence intervals using the Katz et al (1978) method as follows

Letting $T = (X/m)/(Y/n)$, then the random variable $ln(T)$ is approximately normally distributed with approximate mean $ln(\theta)$ and estimated variance

$\hat{\sigma}^2 = ((1/x) - (1/m)) + ((1/y) - (1/n))$.

the approximate two sided $1 - \alpha/2$ confidence interval for θ is given by

$\{t * exp(-Z_{1-\alpha/2}) * \hat{\sigma}, t * exp(Z_{1-\alpha/2}) * \hat{\sigma}\}$,

where $Z_{1-\alpha/2}$ is the $1-\alpha/2$ percentile of the standard normal distribution, and t is the observed value of T

Appendix 3: Prevalence of ever testing for HIV by testing venue

Characteristic	Overall	HIV test taken in			
		Health Facility		Community	
	N	n	Weighted percentage	n	Weighted percentage
**Total**	11,685	8,978	77.1	2,707	22.9
**Gender**					
Male	4,038	2,582	64.2	1,456	35.8
Female	7,647	6,396	84.0	1,251	16.0
**Age**					
15-19	1,498	953	65.1	545	34.1
20-29	4,789	3,894	81.2	895	18.8
30-39	3,422	2,716	79.6	706	20.5
40-49	1,976	1,415	71.7	561	28.3
**Education Level**					
No Education	1,216	1,007	82.3	209	17.7
Primary	6,406	5,003	78.3	1,403	21.7
Secondary+	4,063	2,968	73.7	1,095	26.3
**Marital status**					
Never married	2,159	1,301	61.4	858	14.5
Married/Cohabiting	8,169	6,609	80.8	1,560	19.2
Previously married	1,357	1,068	79.8	289	20.2
**Number of sexual partners in last 12 months**					
0	1,957	1,252	65.4	705	34.6
1	8,649	6,980	80.6	1,669	19.4
2+	1,079	746	69.9	333	30.0
**Currently working**					
No	2,894	2,219	77.5	675	22.5
Yes	8,791	6,759	77.0	2,032	23.0
**Distance to nearest HF**					
<2	2,441	1,920	78.9	521	21.1
2-5	4,823	3,696	77.0	1,127	23.0
5+	4,009	3,040	76.0	969	24.0
Don't Know	412	322	77.0	90	23.0
**Area of residence**					
Rural	8,870	6,845	77.2	2,025	22.8
Urban	2,815	2,133	76.7	682	23.4
**Region**					
Central 1	1,151	889	77.2	262	22.8
Central 2	1,177	861	72.4	316	27.6
Kampala	1,464	1,068	72.2	396	27.8
East Central	1,013	722	72.5	291	27.5
Mid-Eastern	901	732	81.0	169	19.0
North East	1,134	875	75.4	259	24.6
West Nile	1,220	896	72.8	324	27.2
Mid Northern	1,394	1,107	79.4	287	20.6
South Western	1,024	853	83.6	171	16.4
Mid-Western	1,207	975	80.5	232	19.5

Note: Coverage proportions are weighted using population survey weights

Appendix 4: Factors associated with HIV positive among those tested in the 12 months preceding the survey

	Overall				Tested in Health facility		Tested in Community	
**Characteristic**	**cOR**	**(95% CI)**	**aOR**	**(95% CI)**	**aOR**	**(95% CI)**	**aOR**	**(95% CI)**
**Gender (Reference group: Male)**								
Female	1.05	(0.85, 1.29)	1.16	(0.92, 1.47)	1.00	(0.77, 1.29)*	1.76	(1.00, 3.10)*
**Age (Reference group: 20-29)**								
15-19	0.67	(0.45, 0.99)*	0.59	(0.40, 0.88)*	0.68	(0.45, 1.03)	0.34	(0.12, 0.96)*
30-39	1.78	(1.43, 2.22)*	1.56	(1.25, 1.94)*	1.66	(1.31, 2.10)*	1.03	(0.59, 1.80)
40-49	2.45	(1.88, 3.19)*	1.96	(1.51, 2.55)*	2.09	(1.58, 2.77)*	1.49	(0.82, 2.71)
**Education Level ((Reference group: Primary)**								
No Education	1.03	(0.76, 1.40)	0.82	(0.61, 1.11)	0.88	(0.64, 1.20)	0.60	(0.28, 1.37)
Secondary	0.59	(0.48, 0.75)*	0.66	(0.52, 0.83)*	0.61	(0.47, 0.78)*	0.93	(0.64, 1.61)
**Marital status (Reference Group: Married/Living together**								
Never married	0.60	(0.43, 0.85)*	0.74	(0.50, 1.11)	0.85	(0.55, 1.31)	0.46	(0.19, 1.15)
Previously married	3.26	(2.53, 4.19)*	2.53	(1.94, 3.28)*	2.76	(2.08, 3.66)*	1.64	(0.88, 3.05)
**Number of sexual partners in 12 months preceding survey (Reference Group: 1) sig 10%**								
0	1.89	(1.46, 2.46)*	1.93	(1.00, 1.75)	1.39	(1.02, 1.90)*	1.28	(0.63, 2.60)
2+	1.31	(0.94, 1.82)	1.36	(0.99, 1.88)	1.15	(0.79, 1.67)	1.98	(1.05, 3.75)**
**Currently working (Reference Group: Not employed)**								
Employed	1.21	(0.96, 1.52)	1.01	(0.81, 1.25)	1.01	(0.79, 1.29)	0.94	(0.54, 1.65)
**Distance to nearest HF (Reference Group: <2Km)**								
2-5	0.72	(0.55, 0.93)*						
5+	1.07	(0.82, 1.39)						
Don't Know	0.89	(0.51, 1.56)						
**Area of residence (Reference Group: Urban)**								
Rural	0.80	(0.63, 1.02)	0.61	(0.44, 0.85)*	0.58	(0.41, 0.82)*	0.90	(0.39, 2.06)
**Region (Reference Group: Central 1)**								
Central 2	0.69	(0.47, 1.03)**	0.68	(0.46, 1.00)**	0.68	(0.45, 1.04)	0.66	(0.25, 1.71)
Kampala	0.66	(0.43, 1.01)**	0.54	(0.33, 0.88)*	0.51	(0.30, 0.87)*	0.75	(0.24, 2.38)
East Central	0.58	(0.35, 0.97)*	0.54	(0.34, 0.86)*	0.58	(0.36, 0.97)*	0.41	(0.15, 1.13)
Mid-Eastern	0.36	(0.20, 0.64)*	0.46	(0.27, 0.78)*	0.38	(0.21, 0.68)*	0.76	(0.30, 1.94)
North East	0.54	(0.34, 0.85)*	0.61	(0.40, 0.94)*	0.54	(0.34, 0.86)*	1.02	(0.44, 2.37)
West Nile	0.38	(0.21, 0.68)*	0.38	(0.22, 0.65)*	0.34	(0.19, 0.62)*	0.51	(0.19, 1.38)
Mid Northern	0.78	(0.51, 1.19)	0.74	(0.48, 1.13)	0.78	(0.50, 1.22)	0.36	(0.13, 1.01)
South western	0.86	(0.56, 1.32)	0.94	(0.61, 1.45)	0.87	(0.55, 1.38)	1.27	(0.49, 3.30)
Mid-western	0.72	(0.46, 1.15)	0.78	(0.49, 1.22)	0.75	(0.46, 1.22)	0.83	(0.31, 2.26)
